# Advanced cryopreservation engineering strategies: the critical step to utilize stem cell products

**DOI:** 10.1186/s13619-023-00173-8

**Published:** 2023-08-02

**Authors:** Xiaohu Wang, Enyu Wang, Gang Zhao

**Affiliations:** Department of Electronic Engineering and Information Science, University of Science and Technology of China, Hefei, 230027 China

**Keywords:** Stem cell cryopreservation, Photothermal rewarming, Electromagnetic rewarming, Microencapsulation, Synergetic ice inhibition

## Abstract

With the rapid development of stem cell-related therapies and regenerative medicine, the clinical application of stem cell products is on the rise. However, ensuring the effectiveness of these products after storage and transportation remains a challenge in the transformation to clinical trials. Cryopreservation technology allows for the long-term storage of cells while ensuring viability, making it a top priority for stem cell preservation. The field of cryopreservation-related engineering technologies is thriving, and this review provides an overview of the background and basic principles of cryopreservation. It then delves into the main bioengineering technologies and strategies used in cryopreservation, including photothermal and electromagnetic rewarming, microencapsulation, and synergetic ice inhibition. Finally, the current challenges and future prospects in the field of efficient cryopreservation of stem cells are summarized and discussed.

## Background

Stem cell medicine plays an essential role in various biomedical fields, such as regenerative medicine, cell therapy, and tissue engineering (Jin 2017; Yamanaka 2020; Bacakova et al. 2018). With the rapid development of stem cell-based medicine, the demand for high-quality stem cell products has become increasingly urgent (Aijaz et al. 2018). Cryopreservation is necessary for the storage, transportation, and application of stem cell products. Therefore, advanced cryopreservation science has emerged as a critical area of focus (Giwa et al. 2017).

Cryopreservation is a technique that involves cooling biomaterials at low temperatures (typically -80℃ or -196℃) for long-term preservation (Nagashima et al. 1995; Rall et al. 1985). During cryopreservation, cellular metabolism and synthesis in living cells are significantly reduced, or even stagnated, which is the fundamental mechanism for achieving long-term preservation of biological specimens (Steponkus et al. 1990). Cryopreserved stem cells can maintain their initial viability and pluripotency after thawing, allowing for further basic research and clinical applications (Khetan et al. 2019; Khetan 2022).

Physical and chemical damage to stem cells mainly occurs during freeze-thawing procedures in the cryopreservation process (He 2011). Currently, cryopreservation is mainly divided into two categories: programmable slow freezing and vitrification (Zhao et al. 2017). The most commonly used programmable slow-freezing method inevitably results in ice crystal formation both intracellularly and extracellularly during the cooling stage, which is the main cause of physical damage (Zhao et al. 2014). As ice crystals form, the concentration of the remaining unfrozen solution increases, which also leads to chemical damage to the cells (Zhao et al. 2014). Vitrification is another well established technique that does not result in ice crystal formation during the cooling stage (Rall and Fahy 1985). However, it has its own limitation, which is devitrification. Devitrification usually occurs during rewarming and can cause ice recrystallization, physical damage, and ultimately, fatal injury to cryopreserved cells (Biggs et al. 2017; He et al. 2018).

With the explosive development of bioengineering technology, innovative biotechnological tools and materials are being applied to cryopreservation to improve  the efficiency and cell viability during freezing and thawing (Diaz-Dussan et al. 2020; Yao et al. 2022; Naqvi et al. 2018). This has led to a revolutionary advancement in the science of cryopreservation. In this review, we introduce the development history and the fundamentals of cryopreservation, and the emerging engineering techniques in cryopreservation, including photothermal rewarming, electromagnetic rewarming, and microencapsulation. Additionally, we discuss synergistic ice inhibition strategies that combining different ice inhibition methods and highlight the current status of stem cell preservation. By reviewing advanced technologies for inhibiting ice injuries during cryopreservation, we aim to inspire new ideas and reveal new insights into advanced high-efficiency cryopreservation science.

## Cryopreservation

In 1949, the discovery of cryoprotective compounds and their efficacy was first reported, with glycerol being identified as an effective agent for sperm cryopreservation (Polge et al. 1949). Cryopreservation is possible due to the inhibited physiological metabolism of organisms at low temperatures (Vecino et al. 2001), and the Arrhenius formula describes the relationship between metabolic activity and temperature. Based on this formula, biological samples can be stored at -196 °C (liquid nitrogen) for hundreds of years as the physiological and metabolic activities of organisms decrease almost to a standstill at this temperature (Mazur 1984). This theoretical basis for long-term storage at low temperatures is applicable to all biological samples.

In 1972, the two-factor hypothesis of cell damage during freezing was proposed based on the quantitative analysis of the amount of freezing and the permeation process of the solution during freezing (Mazur et al. 1972). The hypothesis states that if the cooling rate is too slow, the extracellular solution will preferentially contact the external cold source and freeze. As a result, an osmotic pressure difference will be created between the inside and the outside of the cell, and intracellular water will continue to leak out. The cells will contract strongly, causing cell shrinkage that can destroy cytoskeleton and protein structures, leading to solution damage (Karlsson et al. 1994; Yang et al. 2009; Zhao et al. 2014; Fahy et al. 2004). Additionally, intracellular ice damage can occur if the freezing speed is too fast, preventing the intracellular solution from infiltrating outside the cell (Karlsson et al. 1994). The two-factor hypothesis provides a framework for understanding the damage caused to cells under different cooling rates and has greatly advanced the field of cryobiology.

To mitigate cell damage during cryopreservation, cryoprotectants (CPAs) are commonly used to improve cell survival rates (Sultanbawa et al. 2001; Langer et al. 2006; Xianqing et al. 2015). CPAs can be permeable or non-permeable depending on their ability to pass through the cell membrane (Cabrita et al. 2003). Permeable CPAs such as dimethyl sulfoxide, glycerol, ethylene glycol, and propylene glycol can enter the cell through the membrane and reduce the amount of intracellular free water, thus decreasing the formation of intracellular ice crystals. Non-permeable CPAs like trehalose, polyethylene glycol, glucan, sucrose, and polyvinylpyrrolidone, on the other hand, cannot enter the cell and usually act on the hydrophobic region of the biofilm, modifying its structural plasticity and increasing its tolerance to freezing (McGann 1978).

In recent years, there have been exciting advancements in the discovery of various highly promising cryoprotective materials. Notably, the discovery of antifreeze proteins (AFPs) in organisms exhibiting exceptional ice recrystallization inhibition has significantly enhanced the post-cryopreservation viability of the human embryonic kidney cell line HEK 293T. This improvement was achieved through the introduction of AFP both inside and outside the cells, demonstrating the remarkable potential of AFPs as cryoprotective agents (Sreter et al. 2022). Widely employed in the realm of cryopreservation, these AFPs have found extensive application in studies involving the cryopreservation of sperm, embryos, and ovaries (Robles et al. 2019; Lee et al. 2015).

In stem cell preservation, dimethyl sulfoxide (DMSO) is commonly employed as a cryoprotectant. However, it has been established that DMSO can exhibit cytotoxicity towards cells (Verheijen et al. 2019). Consequently, there has been growing interest in exploring alternative options. Notably, several synthetic polymers with the ability to suppress ice formation have demonstrated tremendous potential in this regard. Wang conducted research using polyvinyl alcohol (PVA) as a protective agent and demonstrated a significant increase in the viability of mesenchymal stem cells (MSCs). In the presence of PVA, MSC viability rose from 71.2% to an impressive 95.4%. This finding highlights the effectiveness of PVA as a cryoprotectant for preserving MSCs (Wang et al. 2011). Recent reports have provided compelling evidence of the utilization of polyampholytes as innovative protective agents. Drawing inspiration from AFPs, these polyampholytes possess a unique molecular structure that encompasses both negatively and positively charged groups on a single chain. This distinctive configuration imparts exceptional properties in terms of inhibiting ice nucleation and delaying the freezing process. Their remarkable ability to prevent ice formation makes them highly promising for cryoprotection applications (He et al. 2017). Matsumura synthesized carboxylated poly-L-lysine (COOH-PLL) and found that rat MSCs cryopreserved with 7.5% PLL showed significantly higher viability compared to cells preserved with 10% DMSO (Matsumura et al. 2009). Furthermore, cryopreservation with PLL did not cause inappropriate differentiation in the stem cells. He successfully cryopreserved hepatocyte spheroids using a macromolecular cryoprotectant (polyampholytes) combined with a DMSO solution. The study revealed that addition of polyampholytes significantly enhanced post-thaw recovery and minimized cryoinjury-associated membrane damage, surpassing the capabilities of DMSO alone (Matsumura and Hyon 2009). In addition, some nanomaterials such as graphene and nanocellulose are also used in cryoprotection research (Bai et al. 2019; Li et al. 2019a).

In addition to inhibiting ice crystal formation, the protective effect of protectants on cells can be achieved through moderate dehydration. Huang demonstrated the combination of extracellular alginate pre-dehydration and sub-zero temperature ice seeding, resulting in high cell viability of fibroblasts, adult stem cells, and erythrocytes after cryopreservation without the use of permeable protectants (Huang et al. 2017). Similarly, Shen utilized permeable cryoprotectant alginate to dehydrate erythrocytes before freezing, followed by the replacement of intracellular water with a low concentration of glycerol (5% or 7.5%). This method successfully cryopreserved a large number of erythrocytes with a high survival rate of nearly 95% through rapid cooling of EP tubes (Shen et al. 2021). Matsumura reported that controlling osmotic pressure to regulate moderate cell dehydration can significantly inhibit intracellular ice crystal formation by using polyampholytes (Matsumura et al. 2021). These promising cryoprotective materials hold significant research and application value. Exploring and harnessing these protective agents can greatly advance the field of multi-scale cryoprotection, paving the way for significant developments in preserving various biological entities at different scales.

Cooling and warming processes are both important for cell survival during cryopreservation. Traditional cryopreservation methods use slow freezing to reduce the formation of ice crystals in cells by controlling the freezing rate of biological samples, thereby reducing damage to the cell membrane (Meyers 2005) and cytoskeleton (Vincent et al. 1992). During cooling, supercooling of the cell solution occurs, leading to the risk of uncontrollable crystallization and subsequent cell death (Zavos et al. 1983). To address this issue, ice seeding techniques have become common practice in reducing supercooling. Mechanical induction, physical field modulation, and ice-nucleating agents are currently utilized for ice seeding (Weng et al. 2017). Mechanical ice seeding involves the contact of a pre-cooled probe with the supercooled solution, inducing rapid ice formation. Precise modulation of electromagnetic and acoustic fields can also be employed for ice seeding. In recent years, there has been rapid development in ice nucleating agents. Various agents, including sand, polysaccharides, bacterial proteins, lipids, and more, have demonstrated the ability to modulate the formation of ice nuclei (Jiang et al. 2021; Murray et al. 2023; Murray et al. 2022; Miles et al. 2022). The use of ice nucleating agents has proven effective in reducing cell damage during cooling and increasing the survival rate of cryopreserved cells (Daily et al. 2023; Huang et al. 2017).

During rewarming, convective rewarming in the water bath is the main method (Zhao and Fu 2017). However, slow freezing still makes it difficult for cells to completely avoid damage from ice crystal formation (Choi et al. 2011). Vitrification preservation, on the other hand, can prevent ice crystal formation and has better cryopreservation efficiency (Chen et al. 2009). Vitrification involves the direct transformation of the liquid phase into a glassy solid without crystallization, which requires higher CPA concentration and ultra-high cooling rates. High CPA concentrations, however, may cause osmotic shock and chemical toxicity to cells, resulting in cytoskeleton deformation (Vincent et al. 1989; Joly et al. 1992), spindle disintegration, and chromosome diffusion (Saunders et al. 1999). Furthermore, water bath convection during rewarming in vitrification preservation may cause devitrification, which can be fatal to biological samples (Zhao and Fu 2017). Devitrification is significantly related to slow rewarming rates (Han et al. 2008; Zhang et al. 2019), and therefore, we need a rewarming method that can increase the rate of rewarming and reduce devitrification to ensure efficient preservation of biological samples.

## Physical field rewarming

### Photothermal rewarming

With the advancement of engineering technology, numerous potential rewarming technologies are being developed. In this section, we focus on photothermal rewarming and electromagnetic rewarming, discussing their development status and future prospects. In recent years, with the vigorous development of nanoscience, nanoparticles (NPs) with excellent photothermal conversion efficiency have been discovered. To achieve rapid rewarming, researchers began to consider combining these NPs with near-infrared light (Bischof et al. 2018).

**Fig. 1:**
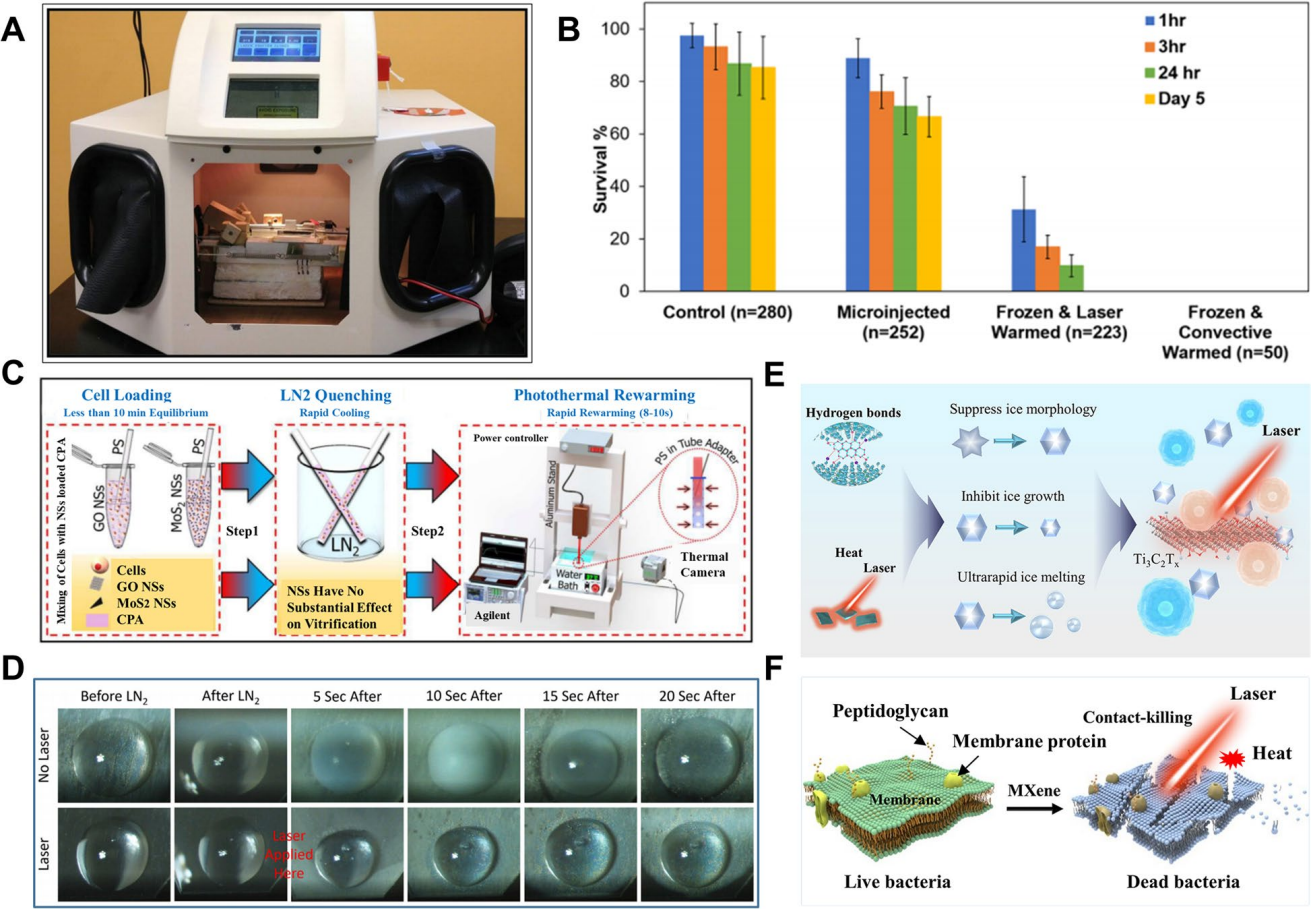
Development of photothermal rewarming. **A** The machine of laser pulse. Reproduced with permission (Jin et al. 2014). Copyright 2014, Elsevier. **B** Comparison of survival viabilities of embryos with four treatments. Reproduced with permission (Khosla et al. 2017). Copyright 2017, American Chemical Society. **C** The cryopreservation procedures based on laser warming. Reproduced with permission (Panhwar et al. 2018). Copyright 2018, Royal Society of Chemistry. **D** Laser rewarming of TiN NPs containing CPA microdroplets. Reproduced with permission (Alvarez et al. 2022). Copyright 2022, Bioengineering and Biotechnology. **E–F** Inhibition Effect of Ti_3_C_2_T_x_ MXene on Ice Crystals Combined with Laser-Mediated Heating. **E** Mechanism of inhibiting ice crystals. **F** The synergetic antibacterial activity of Ti_3_C_2_T_x_ MXene. **E–F** Reproduced with permission (Cao et al. 2022). Copyright 2022, American Chemical Society

In 2014, Jin et al. used India ink as photothermal NPs to significantly inhibit ice crystal formation during rewarming (Jin et al. 2014), and the survival rate of the mouse oocytes was close to 100% after rapid rewarming with laser pulses (Fig. 1A). In convection rewarming, zebrafish embryo preservation did not show good effects due to its large size. To solve this problem, Khosla and his partners considered the use of photothermal rewarming. They injected gold nanorods (GNRs) and propylene glycol into zebrafish embryos for cryopreservation and rewarmed the embryos with 1064 nm laser pulses (Khosla et al. 2017). The results showed that gold nanorods significantly improved embryo viability after hypothermic resuscitation (Fig. 1B). By making full use of the photothermal conversion ability of graphene oxide NPs (Panhwar et al. 2018), Panhwar significantly improved the survival rate of human umbilical vein endothelial cells (HUVECs) after cryopreservation by near-infrared light heating (Fig. 1 C). Moreover, compared with GNRs, titanium nitride (TiN) nanomaterials showed better photothermal ability (Alvarez et al. 2022). TiN nanomaterials were found to provide higher heating rates and temperature uniformity during laser rewarming (Fig. 1D). In addition, TiN has excellent biocompatibility, and human dermal fibroblast (HDF) cells remained at a survival rate of 96% after being co-cultured with TiN solution for 24 h. Rapid melting of ice and reducing devitrification and ice recrystallization were achieved by exploiting the remarkable photothermal conversion properties of Ti_3_C_2_T_x_ (Cao et al. 2022) (Fig. 1E). Moreover, Ti_3_C_2_T_x_ has synergetic antibacterial activity, allowing for stem cell cryopreservation without bacteria (Fig. 1F). The laser rewarming device is relatively simple and is usually used to rewarm small samples due to its limited penetration (Khosla et al. 2019). Furthermore, laser rewarming can be combined with other methods to inhibit ice crystal formation. In the future, photothermal rewarming will not only conduct more research on the biocompatibility of nanomaterials but also pay attention to how to apply it to large-scale biological samples.

### Electromagnetic rewarming

Electromagnetic rewarming is a technique used to inhibit the formation of ice crystals during rewarming by introducing an electromagnetic field. It has been shown to increase heating rates, reduce devitrification, and provide uniform heating (Robinson et al. 2002; Evans 2000; Robinson et al. 1999). Previous studies on microwave rewarming of cryopreserved canine kidneys have revealed limited microwave penetration and a thermal runaway phenomenon in biological tissues during heating (Ketterer et al. 1971; Guttman et al. 1977; Pegg et al. 1978). To overcome the limitations of microwave heating, an open heating system that uses spiral coils to generate low-frequency electromagnetic waves has been developed, enabling uniform heating of the cryoprotectant solution (Ruggera et al. 1990). Closed electromagnetic heating devices have also been developed to limit electromagnetic energy, such as multi-mode resonators (Rachman et al. 1992) and single-mode resonators (Luo et al. 2006). The multi-mode resonator is the superposition of multiple plane waves incident on the sample from different directions, and there are no strict requirements on the size and position of the sample. The single-mode resonator generates standing waves in a specially designed cavity, with strict requirements on the position and size of the sample. Both can improve the heating uniformity, with the latter significantly reducing spatial temperature differences (Wang et al. 2014).

**Fig. 2:**
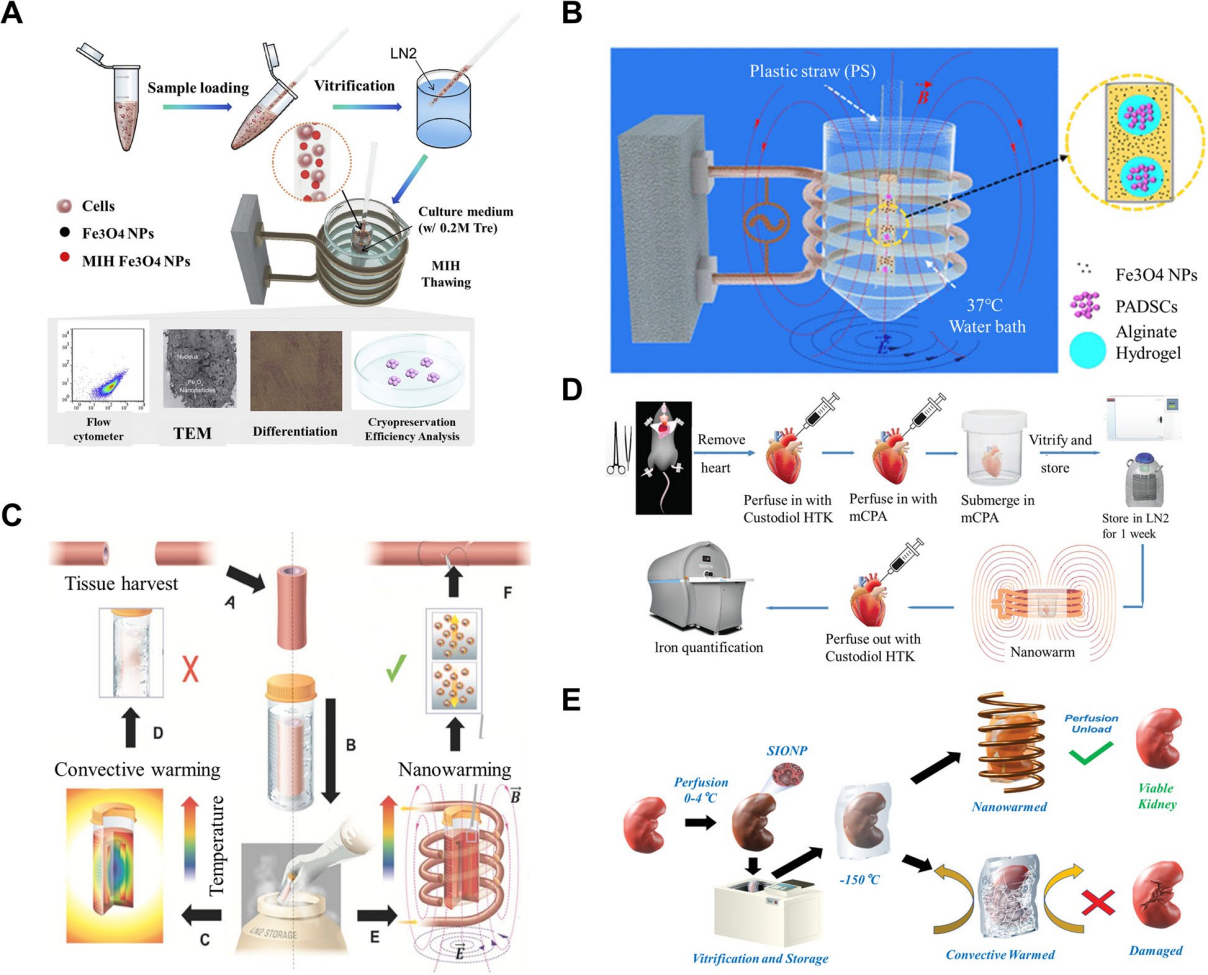
Devitrifification inhibition and uniform heating based on electromagnetic rewarming. **A** The schematic illustration of magnetic warming. Reproduced with permission (Wang et al. 2016). Copyright 2016, Elsevier. **B** Electromagnetic rewarming of stem cells microencapsulated in hydrogels. Reproduced with permission (Liu et al. 2018). Copyright 2018, American Chemical Society. **C** Cryopreservation of blood vessel tissues using magnetic field. Reproduced with permission (Manuchehrabadi et al. 2017). Copyright 2017, American Association for the Advancement of Science. **D** Electromagnetic rewarming of rat heart. Reproduced with permission (Chiu-Lam et al. 2021). Copyright 2021, AAAS. **E** The schematic illustration of complete and ruptured kidneys. Reproduced with permission (Sharma et al. 2021) Copyright 2021, Wiley–VCH

In 2016, magnetic nanoparticles (MNPs) were introduced into an electromagnetic field to revive cryopreserved human umbilical cord blood mesenchymal stem cells (MSCs) (Wang et al. 2016), significantly improving their survival rate after vitrification (Fig. 2A). Several research groups have conducted further studies on MNPs in electromagnetic rewarming, showing that it can preserve intact stem cell microstructures and achieve high immediate cell survival after cryopreservation (Liu et al. 2018) (Fig. 2B). MNPs combined with radio frequency rewarming technology were found to improve the activity of porcine arteries after rewarming (Manuchehrabadi et al. 2017) (Fig. 2C). Mechanical experiments showed that the mechanical properties of nano-magnetothermal rewarming did not change significantly compared with the fresh group. Electromagnetic rewarming of rat hearts significantly improved heating uniformity and reduced devitrification (Chiu-Lam et al. 2021) (Fig. 2D). Perfusion of rat kidneys with MNPs and subsequent electromagnetic rewarming after vitrification preservation significantly inhibited the growth of ice crystals, with the organs showing complete macroscopic structures (Sharma et al. 2021) (Fig. 2E). As research on electromagnetic rewarming deepens, the prospect of cryopreservation for cell tissues and organs becomes more promising.

In summary, electromagnetic rewarming demonstrates significant potential in cryopreservation of biological samples across various scales. However, it is crucial to address certain limitations and disadvantages associated with this technique. One aspect of concern is the introduction of NPs during the process, which may possess cytotoxic properties. Furthermore, incomplete removal of NPs can impact the security of cryopreservation. Additionally, the distribution of NPs within the vascular system may lead to uneven rewarming in avascular or hypovascular sites. These factors warrant careful attention and consideration when implementing electromagnetic rewarming methods.

## Microencapsulation

### Mechanisms and advantages of microencapsulation

Since the first report of hydrogel-encapsulation technology in 1980 (Lim et al. 1980), this method has gained widespread use in various fields such as cryopreservation, transplantation, tissue engineering, and regenerative medicine due to its unique advantages (Zhao et al. 2017; Ramzy et al. 2022; Wilson et al. 2013). Microencapsulated cells can maintain normal physiological activities due to the pores of their encapsulated hydrogels, which can freely pass small molecules such as oxygen, electrolytes, and nutrients, as well as cellular metabolites such as harmful substances like hormones and wastes (Goosen 1992; Olabisi 2015). At the same time, the limited pores can effectively separate larger substances such as pathogenic microorganisms, cells, antibodies, and immunoglobulins from contact with encapsulated cells (Prakash et al. 2005). These advantages enable stem cells to maintain their stemness, persistence, and immunomodulatory properties when transplanted into a specific environment (Zhao et al. 2022; Mao et al. 2019).

Furthermore, microencapsulation can protect cells during cryopreservation. The hydrogel microencapsulation system with a core–shell structure can effectively inhibit the inward growth of ice crystals and protect cells in the microspheres from physical damage caused by ice crystal formation during conventional slow freezing (Kusano et al. 2008). This is one of the main advantages of microencapsulation in cryopreservation. The hydrogel system can also protect cells from chemical damage by effectively buffering the diffusion rate of CPAs, thereby preventing apoptosis caused by transient high concentrations of CPAs (Sarker et al. 2014; Li et al. 2019b). In addition, hydrogel-encapsulation systems can effectively address the major challenge of vitrification preservation, devitrification, by avoiding intracellular ice crystal formation and damage caused by rewarming. Finally, a synergistic ice crystal inhibition strategy involving multiple physical fields combined with microencapsulation, which has different abilities to inhibit ice crystals, plays a crucial role in cryopreservation.

### General methods and relative merits of microencapsulation

Currently, various advanced microencapsulation technologies are being developed. In this section, we focus on the commonly used methods of cell microencapsulation, including conventional microfluidic control, electrostatic spray, and centrifugal microfluidics, as well as their advantages and disadvantages. Since no single method is suitable for all situations, an appropriate method can be selected for microencapsulation according to specific needs.

#### Conventional microfluidics

**Fig. 3:**
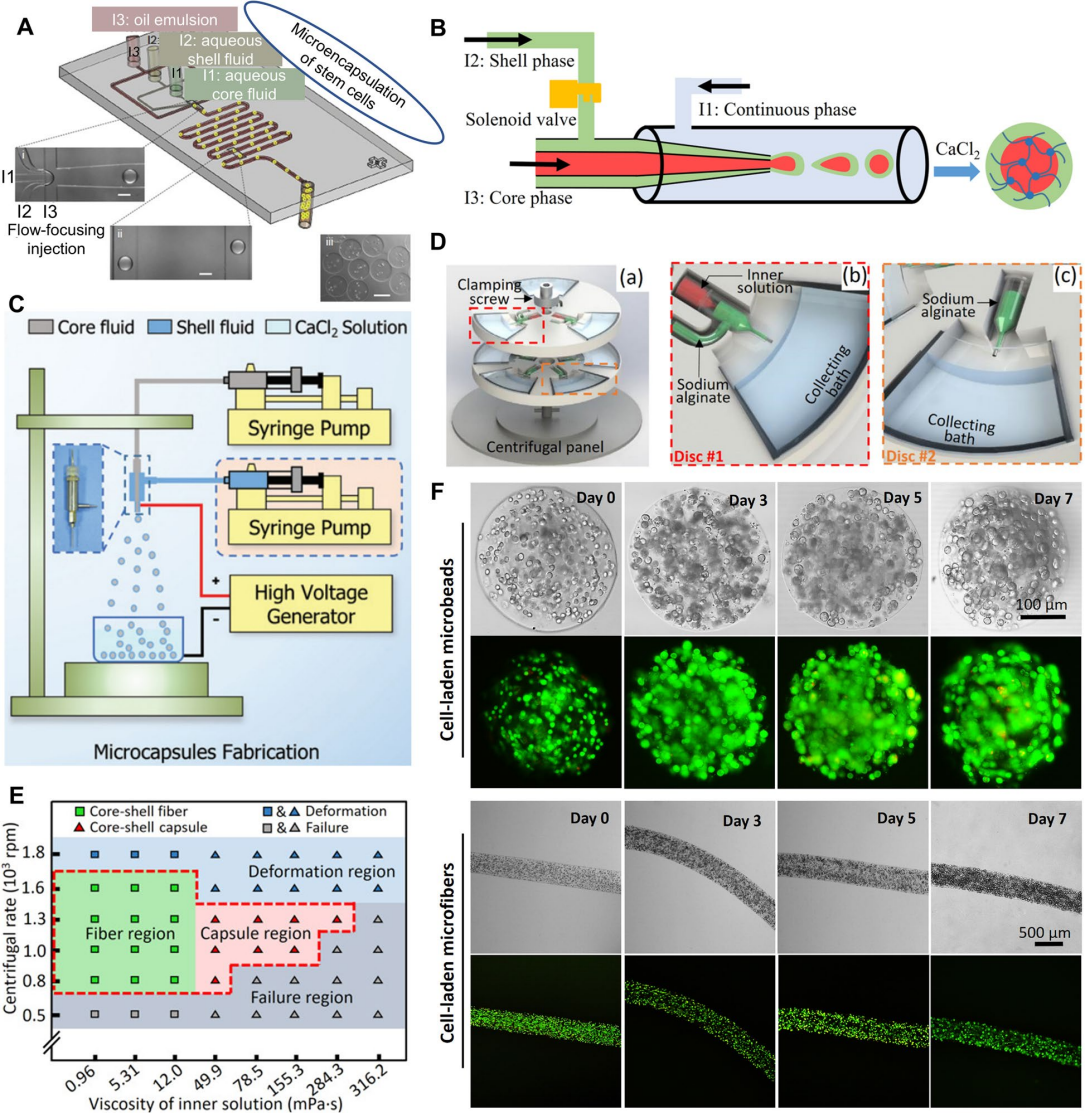
Cell microencapsulation. **A-B** Microfluidic system schematic for fabrication of core–shell capsules. **A** The conventional microfluidic system. Reproduced with permission (Huang et al. 2015). Copyright 2015, Wiley‐VCH. **B** The all-aqueous-phase microfluidic system. Reproduced with permission (Zhu et al. 2019). Copyright 2019, American Chemical Society. **C** The electrostatic spraying system. Reproduced with permission (Zhang et al. 2018). Copyright 2018, Royal Society of Chemistry. **D-F** The centrifugal microfluidic system. **D** Basic structure of the centrifugal microfluidic system. **E** Viscosity and centrifugal rates. **F** 3D culture of simple structured cell-laden microcarriers and cell viability tests. **D**-**F** Reproduced with permission (Cheng et al. 2018). Copyright 2015, Elsevier

Generally, microfluidics can produce more homogeneous microcapsules compared to electrostatic spray and centrifugation (Zhao et al. 2017; Cheng et al. 2018). However, conventional microfluidic control requires high conditions and special facilities, such as clean rooms and skilled researchers (Kang et al. 2014). Additionally, some materials, such as plastic-based microfluidic devices, may not be suitable for long-term or reuse as they are prone to aging or clogging (Zhao et al. 2017). Conventional microfluidics also introduces oil as a carrier phase for the formation of microcapsules (Fig. 3A), leading to problems such as biological toxicity and contamination (Zhu et al. 2019; Cheng et al. 2018). In response to these issues, researchers have made some improvements. For instance, a controllable all-aqueous-phase microfluidics method was presented (Fig. 3B) for generating stable and continuous core–shell microcapsules (Zhu et al. 2019). Moreover, a tube-in-tube capillary microfluidic device was developed to encapsulate stem cells in core–shell microcapsules without using toxic acid or oil and can be repeatedly used in the long term (Zhao et al. 2017).

#### Electrostatic spraying

Electrostatic spraying is a commonly used method of encapsulation (Fig. 3C). Significant advances have been made in the fields of tissue engineering and cryopreservation, including bone repair (Yang et al. 2021), cardiac injury repair (Choe et al. 2019), and long-term storage (Gryshkov et al. 2021, 2014; Lu et al. 2017; Zhang et al. 2018) after stem cells were encapsulated using electrostatic spraying. The mechanism involves adjusting various parameters in the system, such as voltage, flow rate, needle gauge, working distance, and biomaterial properties (e.g., concentration/viscosity, conductivity), to create a jet that rapidly produces particles and fibers (Naqvi et al. 2016). This technology is more efficient than conventional microencapsulation methods (Naqvi et al. 2016). However, it still faces several challenges. For example, the production of some polydisperse microcarriers is inevitable, high operating voltage may damage cells during encapsulation, and the size distribution of produced microcapsules might be wide (Zhao et al. 2017; Cheng et al. 2018). Nevertheless, electrostatic spraying requires high operational skills and complicated facilities and equipment, which may become another limitation to researchers.

#### Centrifugal microfluidics

A recent report introduced an ultra-simple centrifugal microfluidic system that can produce core–shell capsules/fibers (Fig. 3D), generate core–shell capsules/fibers by adjusting the centrifugal force and the viscosity of the internal solution (Fig. 3E), and use them for cryopreservation and 3D culture of cells (Fig. 3F) (Cheng et al. 2018). Compared with conventional microfluidics, centrifugal microfluidics has unique advantages. First, it does not require high operational skills or complex experimental facilities (only a centrifuge). Second, the system does not require a carrier phase (oil), which avoids washing and possible contamination. Third, the centrifugal device has a simple structure, and the solution and cells can completely enter the collection bath under centrifugation to avoid waste. However, this device also has some limitations. The size of microspheres and microfibers produced cannot be precisely controlled due to the alteration of centrifugal force. Therefore, investigators should choose appropriate encapsulation methods based on their specific requirements.

## Synergetic ice inhibition

Both the introduction of physical fields in the rewarming process and the use of microencapsulation in the preservation process offer new ways to enhance the efficiency of cryopreservation. In addition, the development of materials with significant ice-inhibition capabilities and trehalose delivery technology effectively promotes cell preservation. The synergistic ice inhibition combines these advanced engineering strategies based on their properties, significantly suppressing ice formation during cryopreservation and improving the survival rate of samples.

**Fig. 4:**
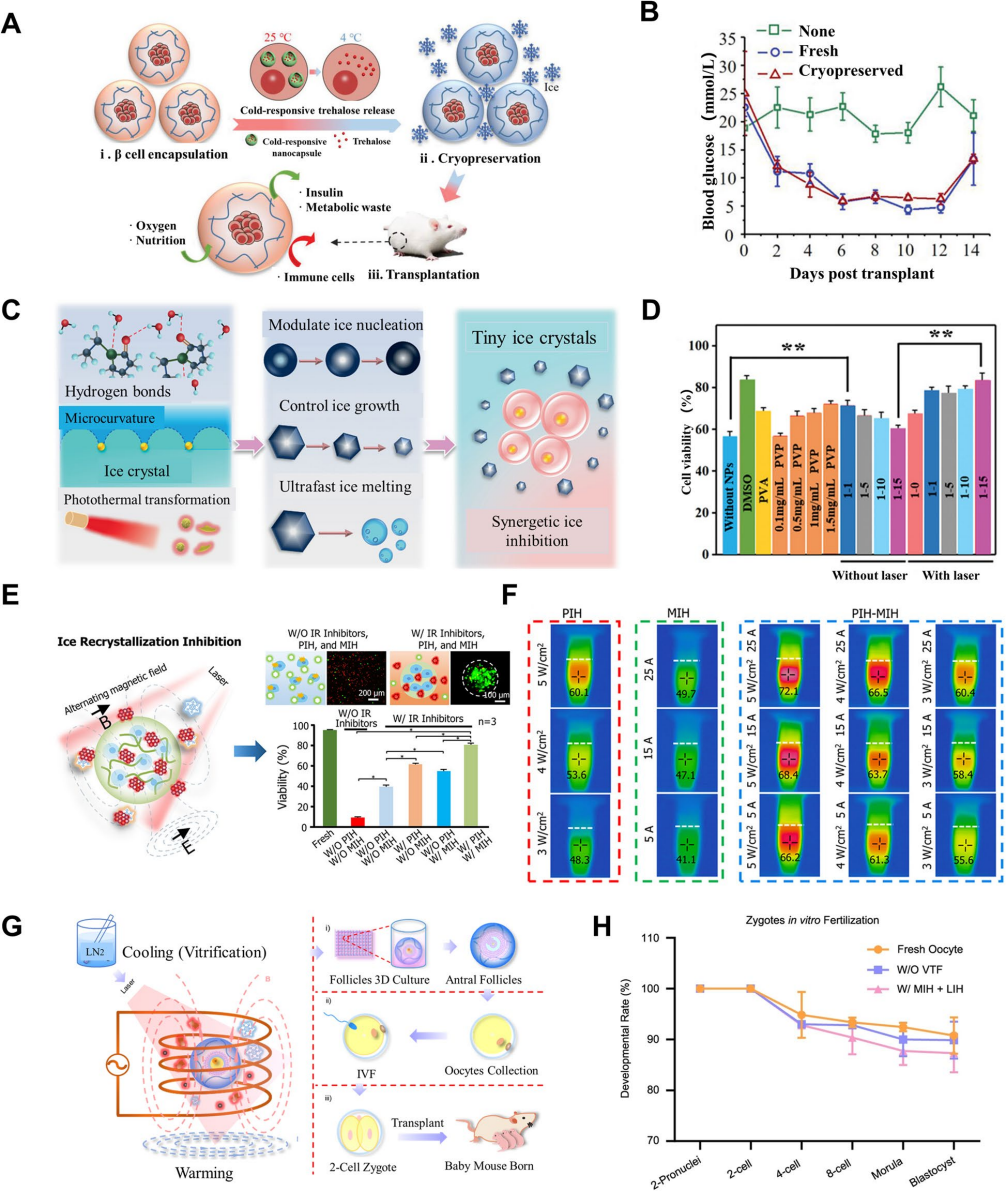
Synergistic ice inhibition for cryopreservation. **A-B** Cryopreservation of pancreatic islet β cells. **A** The schematic illustration of encapsulation and alginate delivery. **B** Blood glucose changes after transplantation. (A-B) Reproduced with permission (Cheng et al. 2019). Copyright 2019, Wiley–VCH. **C-D** Synergistic ice inhibition improves cell survival. **C** Schematic illustrations of synergetic ice inhibition mechanisms. **D** Different cell viability. (C-D) Reproduced with permission (Chang et al. 2021). Copyright 2021, Wiley–VCH. **E–F** MSCs freezing strategy. **E** The schematic illustration of freezing and rewarming process. **F** The effect of different physical fields. (E–F) Reproduced with permission (Cao et al. 2019). Copyright 2019, American Chemical Society. **G-H** Vitrification cryopreservation of mouse preantral follicles. **G** Schematic diagram of follicle cryopreservation. **H** Embryo development rate. **G**-**H** Reproduced with permission (Tian et al. 2022). Copyright 2022, Springer Nature

Intracellular ice damage is a significant risk factor in cell cryopreservation (Poisson et al. 2019), and trehalose is an excellent ice-suppressing protective agent that has been extensively studied in cell preservation (Sharp et al. 2013). However, as a non-permeable protective agent, it lacks the ability to enter cells. Cheng et al. introduced trehalose into pancreatic islet β cells and combined hydrogel encapsulation and physical field rewarming to achieve efficient preservation of islet β (Cheng et al. 2019). In vivo transplantation experiments showed that the preserved cells still had the ability to lower blood glucose (Fig. 4 A and B). Based on the synergistic ice inhibition approach, Chang et al. prepared WSe_2_-PVP nanomaterials with ice inhibition and photothermal capabilities for cell preservation (Chang et al. 2021). The results showed that this strategy significantly increased the cell survival rate (Fig. 4 C and D). Cao et al. used the magneto- and photothermal dual response capability of GO − Fe_3_O_4_ nano-composites, combined with hydrogel encapsulation for cryopreservation of stem cells, which significantly increased the warming rate and stem cell survival rate (Cao et al. 2019) (Fig. 4 E and F). To achieve low-concentration vitrification preservation of mouse preantral follicles, Tian et al. combined hydrogel encapsulation and physical field rewarming, which greatly increased the warming rate and inhibited devitrification (Tian et al. 2022). The results showed that the group with multiple physical fields used in concert exhibited higher cell survival rates and embryo development rates (Fig. 4 G and H). In summary, the synergistic ice inhibition strategy has shown great potential in inhibiting ice crystal formation and improving stem cell survival. With the continuous advancement of engineering technology, it is hopeful that biological samples of larger size can be successfully preserved.

## Stem cell preservation

Stem cells are extensively utilized in cell-based therapies and regenerative medicine due to their remarkable potential for self-renewal and differentiation into various cell types (Niwa et al. 1998; Dixon et al. 2014). However, when subjected to long-term culture conditions, stem cells can be prone to genotypic drift, chromosomal abnormalities, phenotypic instability, and contamination. To address this, cryopreservation of stem cells is crucial to meet the growing demand for stem cell products (Ben-David et al. 2011).

In clinical practice, slow freezing is the commonly employed method for stem cell preservation. It offers simplicity in operation and manageable costs, but it unavoidably causes ice-induced damage to the cells. Vitrification, on the other hand, enables ice-free preservation but requires high concentrations of CPAs, which can be somewhat cytotoxic (Calabrese et al. 2010). To enhance the efficiency of stem cell cryopreservation, various preservation methods have been explored, such as photothermal rewarming, electromagnetic rewarming, and microencapsulation (Table 1). These approaches aim to improve the overall preservation outcomes and minimize the adverse effects associated with traditional cryopreservation methods.

**Table 1:** Application of engineering technology in stem cell preservation

Engineering Strategy	Genus	Stem cell types	Advantages
Photothermal rewarming	Human	hUC-MSCs	High vitality
Electromagnetic rewarming	Porcine Human	pADSCs hUC-MSCs hiPSCs	High vitality Large volume preservation
Microencapsulation	Mice Human	mESCs hADSA hBMSCs hUC-MSCs	High vitality Large volume preservation High biocompatibility Widely used

Photothermal rewarming techniques utilizing materials such as soft liquid metal nanoparticles and Ti_3_C_2_T_x_ (Cao et al. 2022) showed significant improvements in preserving human bone marrow mesenchymal stem cells and MSC viability. Laser heating with GNRs achieved high survival rates for human umbilical cord blood stem cells (Zhan et al. 2021). Wang et al. achieved efficient electromagnetic rewarming-assisted vitrification of human umbilical cord blood mesenchymal stem cells, a groundbreaking milestone in stem cell cryopreservation (Wang et al. 2016). Hydrogel encapsulation combined with low concentration cryoprotectant vitrification demonstrated a survival rate of over 80% for porcine adipose stem cells (pADSC) (Liu et al. 2018). Ito et al. successfully preserved human induced pluripotent stem cells (hiPSCs) on a large scale (20 ml) using electromagnetic rewarming, showing promising industrial potential (Ito et al. 2020). These advancements highlight the potential of electromagnetic and photothermal rewarming in enhancing stem cell preservation methods.

In contrast to the aforementioned emerging technologies, hydrogel encapsulation has found extensive applications in biomedicine. The use of alginate microcapsules for cell encapsulation was first demonstrated in 1980 (Lim et al. 1980), and applied to cryopreservation in 1993 (Dixit et al. 1993). In 1994, microencapsulated islets were transplanted into a diabetic patient, successfully maintaining normal blood glucose levels for 9 months, marking the first clinical use of microencapsulation (Soonshiong et al. 1994). Hydrogel microcapsules offer exceptional properties such as ice suppression, high biocompatibility, and anti-glassing inhibition, making them valuable for stem cell cryopreservation.

Studies have shown that alginate-encapsulated mesenchymal stem cells retain high viability and maintain their multidirectional differentiation potential after cryopreservation (Pravdyuk et al. 2013). Huang et al. reported successful low concentration cryoprotective agent (CPA) vitrification of mouse embryonic stem cells (mESCs) and human adipose stem cells (hADSC) with the assistance of alginate hydrogel microcapsules, resulting in a significant increase in cell viability (Huang et al. 2015). Hydrogel microencapsulation has also demonstrated efficient cryopreservation of other stem cell types, such as cord blood mesenchymal stem cells (MSCs) and bone marrow MSCs (Pravdyuk et al. 2013; Katsen-Globa et al. 2014). Moreover, alternative hydrogel materials including hyaluronic acid hydrogels (Khetan and Corey 2019) and polymeric peptide hydrogels (Anderson et al. 2011) have shown great potential in stem cell cryopreservation. Microencapsulation serves as a foundational technology that can be combined with other advanced engineering strategies to achieve optimal preservation of stem cells.

## Conclusions and perspectives

This review highlights the significant progress that has been made in the engineering technology of cryopreservation of stem cells. The preservation of stem cell function and differentiation ability after long-term cryopreservation and resuscitation is critical to realizing the clinical applications of stem cell-related tissue engineering and regenerative medicine. We have discussed the primary cryodamage mechanism of stem cells during the cooling-warming process of cryopreservation and introduced a series of advanced ice inhibition engineering technologies, including photothermal, magnetothermal rewarming, cell encapsulation, and synergetic ice inhibition. Efficient stem cell cryopreservation is crucial to meeting the urgent needs of subsequent stem cell-related regenerative medicine and bioengineering applications through the engineering ice inhibition strategy.

In a broader sense, devitrification during stem cell rewarming remains the main challenge of vitrification preservation. At present, advanced engineering strategies for stem cell cryopreservation are still under further development. Specifically, combining multiple advanced engineering technologies, such as applying nanomaterials with ice inhibition and conversion ability (Cao et al. 2022), using magnetothermal rewarming microencapsulation technology (Cheng et al. 2019), or utilizing photo- and magnetoresponsive materials that convert light and magnetic energy into heat in combination with encapsulation technology (Cao et al. 2019), could further avoid permeation damage and achieve low-CPA and high-grade vitrification of biocomposites.

Although substantial progress has been made, the implementation of laboratory-level processes remains a significant limitation for clinical applications. Future research should focus on the realization of manufacturing under relevant Good Manufacturing Practices (GMPs) through these strategies to meet current application requirements. These engineering strategies offer various advantages in stem cell cryopreservation, with hydrogel encapsulation playing a significant role in industrial applications. Hydrogel scaffolds loaded with stem cells have gained prominence in cell therapy, tissue engineering, and regenerative medicine. The incorporation of stem cells into hydrogel scaffolds enhances their storage and utilization, especially if the hydrogel scaffolds improve cell survival during cryopreservation. Additionally, for large-volume hydrogel stem cell products, the combination with electromagnetic rewarming can further enhance the survival rate. Given the irreplaceable characteristics of stem cells in tissue engineering and regenerative medicine, we believe that advanced and preferable stem cell cryopreservation engineering strategies will keep abreast of emerging demands, promising a thriving future for regenerative medicine.

## Data Availability

Not applicable.

## Abbreviations

CPA: Cryoprotectant
NPs: Nanoparticles
MNPs: Magnetic nanoparticles
GNRS: Gold nanorods
TiN: Titanium nitride
MSCs: Mesenchymal stem cells
mESCs: mouse embryonic stem cells
hiPSCs: human induced pluripotent stem cells
hADSC: human adipose stem cells
pADSC: porcine adipose stem cells
AFPs: antifreeze proteins
PVA: polyvinyl alcohol
